# A Method of Expelling Foreign Objects Taken into the Digestive Tube

**Published:** 1891-05

**Authors:** 


### A METHOD OF EXPELLING FOREIGN OBJECTS TAKEN INTO THE DIGESTIVE TUBE.

(Translated from the Spanish by E. A. P.)

The process actually in practice at Dr. Billroth's clinics is known by the name of Potato Cure, and was indicated by Cameron (of Glasgow) in 1887.

The patient should eat a large quantity of potatoes, which will produce a uniform distention of the intestinal tube and provoke the expulsion of the foreign body by the natural way.

It has been successful in many cases. A weight of two decigrams swallowed by a child, a splinter five decimetres long and three centimetres thick swallowed by a servant, and a thorn, by a young man, all were thus removed.

Billroth thinks that many cases of gastrotomy could be avoided by first trying the potato cure.

A nail has been presented by Dr. Hochenegg to the Society of Doctors of Vienna, as a further proof of Surgeon Billroth's method. This nail was swallowed and after nine days discharged.

Professor Albert had performed a notable gastronomic operation on the same patient in 1884, for the purpose of extracting a nail of the same dimensions. Difficulty was experienced then to find it, although the stomach had been opened.

—*Revista Homœopatica*, August, 1890.

